# Mercury intoxication and ophthalmic involvement: An update review

**DOI:** 10.3389/ftox.2023.1148357

**Published:** 2023-03-31

**Authors:** Cristian de los Santos, J. Carlos Pastor, Margarita Calonge

**Affiliations:** ^1^ Institute of Applied Ophthalmobiology (IOBA), Universidad de Valladolid, Valladolid, Spain; ^2^ Centro en Red de Medicina Regenerativa y Terapia Celular. Junta de Castilla y León, Valladolid, Spain; ^3^ CIBER-BBN, Carlos III National Institute of Health, Madrid, Spain

**Keywords:** corneal sensitivity, dry eye disease, mercury intoxication, ocular surface, ophthalmic involvement, optic nerve, retina, visual function alterations

## Abstract

Human intoxication after mercury exposure is a rare condition that can cause severe damage to the central nervous, respiratory, cardiovascular, renal, gastrointestinal, skin, and visual systems and represents a major public health concern. Ophthalmic involvement includes impaired function of the extraocular muscles and the eyelids, as well as structural changes in the ocular surface, lens, retina, and optic nerve causing a potential irreversible damage to the visual system. Although, there are many pathways for poisoning depending on the mercury form, it has been suggested that tissue distribution does not differ in experimental animals when administered as mercury vapor, organic mercury, or inorganic mercury. Additionally, visual function alterations regarding central visual acuity, color discrimination, contrast sensitivity, visual field and electroretinogram responses have also been described widely. Nevertheless, there is still controversy about whether visual manifestations occur secondary to brain damage or as a direct affectation, and which ocular structure is primarily affected. Despite the use of some imaging techniques such as *in vivo* confocal microscopy of the cornea, optical coherence tomography (OCT) of the retina and optic nerve, and functional tests such as electroretinography has helped to solve in part this debate, further studies incorporating other imaging modalities such as autofluorescence, OCT angiography or adaptive optics retinal imaging are needed. This review aims to summarize the published structural and functional alterations found in the visual system of patients suffering from mercury intoxication.

## 1 Introduction

Mercury is a toxic metal that exists in three forms with different toxicological properties, as elemental or metallic mercury (mercury liquid and mercury vapor) or as inorganic (mercury salts) and organic compounds (methylmercury and ethylmercury) when combined with other elements (Park and Zheng, 2012; Fowler and Zalups, 2022), and it is considered by the World Health Organization (WHO) as one of the top 10 chemicals or groups of chemicals of major public health concern (World Health Organization, 2017). A significant incident occurred in Minamata, Japan, between 1932 and 1968, where a factory dumped waste liquid with high concentrations of methylmercury in the bay which was rich in fish and shellfish, the primary food sources for local and other areas residents, affecting at least 50,000 people and causing neurological symptoms in over 2,000 people (hence the name Minamata disease) (World Health Organization, 2017). Later in 1972, over 6,000 people in Iraq developed methylmercury poisoning from eating baked grain bread treated with methylmercury-based fungicide (Fowler and Zalups, 2022; Posin et al., 2022).

There are many pathways for intoxication after mercury exposure, including ingestion of contaminated seafood, contact with broken mercury-containing devices such as thermometers, barometers and electrical switches, or inhaling mercury vapor from dental amalgam (which are currently less and less likely because their manufacture has been prohibited) (Fisher, 2003; World Health Organization, 2017; Fowler and Zalups, 2022; Posin et al., 2022). However, most human mercury intoxication occurs in occupational settings when workers inhale odorless and colorless elemental mercury vapors, in mercury and artisanal or small-scale gold mining, physics and pharmaceutical laboratories and some industrial processes such as zinc-mercury amalgam, coal-fired power and chloroalkali plants, paint factories, non-ferrous and ferrous metal production, and in fluorescent lamp, batteries and other instruments manufacturing (Fisher, 2003; Rustagi and Singh, 2010; UN Environment, 2019; Fowler and Zalups, 2022). These mercury vapors are absorbed up to 80% through the lungs with rapid diffusion to the blood and later distribution throughout the body. In contrast, inorganic mercury is absorbed mainly in the gastrointestinal tract in about 2%–38%, while methylmercury when ingested is almost 100% absorbed in the duodenum, then in the blood combines with glutathione and other amino acids or peptides (Hong et al., 2012; Fowler and Zalups, 2022; Posin et al., 2022).

Due to the lipophilic nature of elemental and organic mercury, both can cross the blood-brain barrier. Then, they are oxidized by the hydrogen peroxide-catalase pathway to an inorganic divalent form with poor lipid solubility, and therefore, accumulate for several years in the brain, interrupting cellular enzymes and proteins systems, and causing neurotoxicity (Fisher, 2003; Posin et al., 2022).

Peripheral nerve function, renal function, immune and endocrine systems, and muscle function may also be affected by the three forms (Fowler and Zalups, 2022). To reduce these adverse effects, a global agreement named Minamata Convention on Mercury was adopted in 2013 and entered into force in 2017 to take actions to protect human health and environment from anthropogenic release of mercury (UNEP, 2019). Mercury levels in blood are useful after short-term and high-level exposure, whereas mercuric values in urine mercury is the ideal biomarker for long-term exposure to both elemental and inorganic mercury (Park and Zheng, 2012).

Eye and visual pathway damage have been reported given the fact that the retina and the optic nerve are specialized extensions of the central nervous system (CNS) (London et al., 2013). Furthermore, mercury intoxication may also cause damage to the corneal nerves as the cornea is the most densely innervated tissue in the body. In this review we focus on ophthalmic involvement due to mercury intoxication and summarize the clinical experience of our center about this topic based on 29 workers suffering acute and subacute exposure to mercury vapor in an aluminum manufacturing industry that were studied and followed by the Institute of Applied Ophthalmobiology (IOBA), University of Valladolid, Spain (Cañadas et al., 2021; Pastor-Idoate et al., 2021).

## 2 Materials and methods

Articles were sourced using PubMed database with the following terms: “color vision”, “contrast sensitivity”, “cornea”, “exposure”, “eye”, “glaucoma”, “heavy metal”, “intoxication”, “lens”, “mercury”, “ocular alterations”, “ocular manifestations”, “ocular surface”, “ophthalmological findings”, “optical coherence tomography”, “optic nerve”, “poisoning”, “retina”, “toxicity”, “visual alterations” and “visual evoked potentials”. We completed the selection of pertinent literature until the inception of the manuscript (October 2022) based on title, abstract and full content information.

## 3 Results

### 3.1 Ocular surface disease and anterior segment alterations

The lacrimal functional unit, a term first introduced by Stern et al., is a unit composed of the ocular surface (corneal, conjunctival and limbal epithelium plus the overlying tear film), all tear-producing glands and cells, in addition to the immune cells and nervous fibers that work together to maintain the health of the cornea (Stern et al., 2004), which is the major refractive surface of the visual system and the most sensitive tissue in the body, being densely innervated at its external layers by the first division (ophthalmic) of the trigeminal nerve.

Neurotoxicity induced by mercury may target this rich innervation as previously reported by Sabelaish and Hilmi who described loss of corneal sensation in most affected patients after subchronic organomercury poisoning, however, no objective test was performed to confirm this finding (Sabelaish and Hilmi, 1976). Nevertheless, Cañadas et al., from our group, evaluated 22 male workers who were accidentally exposed to mercury vapor for 14 consecutive days and described diminished corneal sensitivity as well as decreased nerve density and branch density of sub-basal corneal nerves, and reduced density of dendritic cell in corneal stroma determined by non-contact Belmonte gas esthesiometry and *in vivo* confocal microscopy, respectively, and thus impairing both nerve function and nerve regenerative activity (Cañadas et al., 2021). Most of these patients referred dry eye symptoms, mostly severe, using the Ocular Surface Disease Index questionnaire (OSDI) and showed increased tear osmolarity compared to control healthy subjects, however, no alteration in tear quality and ocular surface integrity was found. Tear production was not significantly affected in those workers, although 8 patients showed low lysozyme tears levels, particularly with some elevated cytokines in tears, such as interleukin (Il)-6, lL-12p70, regulated on activation normal T-cell expressed and secreted (RANTES), and vascular endothelial growth factor (VEGF) and with high urine mercury levels. So far, this is the only report on ocular surface disease in subacute mercury intoxication in humans and taking this evidence together, a primary neurogenic inflammation mechanism triggering a proinflammatory cascade of cytokines may explain ocular surface disease in mercury poisoning (Cañadas et al., 2021; Pollard et al., 2019; Yang et al., 2020).

Less frequently, band keratopathy, mercury deposits on the corneal stroma and anterior capsule of the lens (mercurialentis) have also been reported in some cases of chronic mercury intoxication (El-Sherbeeny et al., 2006). In addition, Korbas et al. in studies with zebrafish larvae (*Danio rerio*), suggested that methylmercury may accumulate in the secondary fiber cells of the lens after reaching high intraocular levels by being able to cross the blood-aqueous barrier (Korbas et al., 2008; Korbas et al., 2013). Furthermore, Domínguez-Calva et al. showed that mercury has a cataractogenic potential by inducing non-amyloid aggregation of human lens proteins (γC and γS crystallins proteins) (Domínguez-Calva et al., 2018).

### 3.2 Retina, optic nerve, and visual alterations

Accumulation of mercury in the retinal pigment epithelium, inner plexiform layer, ganglion cells and vessel walls of the inner retina and of the optic nerve was described by Warfvinge and Bruun in squirrel monkeys up to 3 years after mercury vapor exposure (Warfvinge and Bruun, 2000). Similarly, Phamphlett et al. found that mercury may appear in fetal retinal ganglion cells, optic nerve glial cells, peripapillary retinal pigment epithelium, and endothelial cells of mice after prenatal exposure to mercury vapor (Pamphlett et al., 2019). Korbas et al. demonstrated that methylmercury may target both optic nerve (Korbas et al., 2008) and outer segments of photoreceptors cells (Korbas et al., 2013) in zebrafish larvae. Additionally, it has been suggested that even though there are three forms of mercury, retinal and optic nerve distribution seems not to differ in experimental animals when administered as mercury vapor, organic mercury, or inorganic mercury (Pamphlett et al., 2019).

Visual impairment due to mercury toxicity may occur as a direct eye damage as demonstrated by the IOBA’s Retina group and other researchers (Bridges et al., 2007; Korbas et al., 2013; Pastor-Idoate et al., 2021) in addition to visual cortex injury (da Costa et al., 2008; Saldana et al., 2006; Ventura et al., 2004; Ventura et al., 2005; Yorifuji et al., 2013), causing night vision dysfunction, decreased color vision and contrast sensitivity, central visual impairment, visual field (VF) defects such as concentric constriction, and optic atrophy (El-Sherbeeny et al., 2006; Bridges et al., 2007; Pastor-Idoate et al., 2021; da Costa et al., 2008; Saldana et al., 2006; Ventura et al., 2004; Ventura et al., 2005; Yorifuji et al., 2013; Cavalleri et al., 1995; Cavalleri and Gobba, 1998; Urban et al., 2003; Rodrigues et al., 2007; Fillion et al., 2011; Dos Santos Freitas et al., 2018; Feitosa-Santana et al., 2008; Feitosa-Santana et al., 2007; Feitosa-Santana et al., 2018; Barboni et al., 2008).

Some studies have reported retinal pigment epithelium (Bridges et al., 2007) and photoreceptor damage (Korbas et al., 2013). Recent evidence by Pastor-Idoate et al., from our group, showed a primary involvement in electroretinogram (ERG) of both the inner (oscillatory potentials) and outer retina, mainly reduced scotopic rod response, in a long-term group of affected patients (Pastor-Idoate et al., 2021). Nevertheless, 30-Hz flicker, single flash cone response and multifocal ERG and pattern ERG alterations also appeared when deeper and more extensive VF defects developed, suggesting that cone dysfunction and ganglion macular cells damage can occur secondarily, thus causing color vision impairment, mainly in the blue-yellow range using Roth 28 Hue test. In addition, prolonged latencies, and reduced amplitudes of P100 in visual-evoked potentials compared to controls were found when severe VF was altered (Pastor-Idoate et al., 2021), as previously reported (El-Sherbeeny et al., 2006). In summary, although neurologic and visual pathway involvement was clear, there were also data suggesting the existence of a direct functional retinal damage and retinal participation in mercury poisoning.

Ekinci et al. reported in 31 industrial mercury battery workers blue-yellow color vision impairment but reduced retinal nerve fiber layer thickness (RNFLT) and choroidal thickness (CT) (Ekinci et al., 2014) on optical coherence tomography (OCT), data not confirmed by Pastor-Idoate et al. (Pastor-Idoate et al., 2021) who found normal RNFLT, CT, and central retinal thickness. Additionally, Bilak et al. demonstrated reduced volumes of the inner plexiform and ganglion cell layers on OCT in patients exposed to mercury from amalgam dental fillings compared to controls, however, RNFLT and CT decreases were neither significant nor clinically relevant (Bilak et al., 2019). An important aspect of the case series by Bilak et al. and Ekinci et al. is that they were done in patients chronically exposed to mercury and ocular electrophysiologic studies were not performed in contrast to the clinical analysis done by Pastor-Idoate et al. in patients with acute/subacute exposure to mercury. Blood concentrations may have been lower and evidently exposure times were different.

On the other hand, Cavalleri et al. (1995) and Jedrejko and Skoczyńska (2011) also observed color vision alteration in the blue-yellow range in workers exposed to mercury vapor, whereas Ventura et al. (2005) and Feitosa-Santana et al. (2008) found both blue-yellow and red-green alterations in patients with chronic mercury vapor intoxication, suggesting alterations in both the retina and the optic nerve. Lacerda et al. investigated two Amazonian populations, 10 Riverines exposed to organic mercury by eating fish and 34 gold-miners exposed to mercury vapor, and described that both groups had similar color vision impairment compared to control groups using Farnsworth–Munsell test, however, visual perimetry impairment was greater in riverines than in gold-miners using Förster perimeter, which may be due to higher exposure to mercury in riverines (Lacerda et al., 2020).

There is no consensus about the reversibility of mercury intoxication. Previous studies described that color vision loss may be reversible (Cavalleri and Gobba, 1998; Urban et al., 2003), however, recent reports strongly suggest irreversible damage in both chronic methylmercury consumption and in workers chronically exposed to mercury vapor (Feitosa-Santana et al., 2007; Feitosa-Santana et al., 2008; Feitosa-Santana et al., 2018). Similarly, Ventura et al. and Costa et al. also found that contrast sensitivity is irreversibly impaired in long-term occupational mercury intoxication (Ventura et al., 2005; Costa et al., 2008).

### 3.3 Other ophthalmic manifestations

Mercury has also been suggested to be linked to glaucoma (Vennam et al., 2020). Ceylan et al. found significantly higher blood mercury levels in 32 patients with pseudoexfoliation syndrome compared to controls (Ceylan et al., 2013). Pseudoexfoliation syndrome is considered a systemic disease characterized by accumulation of extracellular material, named pseudo-exfoliative, in many organs including the eye and orbit, mainly on the anterior lens capsule and/or the pupillary border and which may impair aqueous drainage and thus high intraocular pressure and glaucoma (Plateroti et al., 2015). Pseudoexfoliation origin is not fully established and is more frequent in Northern European countries. Trace elements have been suggested to have roles in its pathogenesis, however no pseudoexfoliation glaucoma association was significantly found in these patients (Ceylan et al., 2013). Similarly, Lee et al. (48) based on data from the Korean National Health and Nutrition Examination Survey (KNHANES) did not find significant associations between blood levels of mercury and open angle glaucoma prevalence (Lee et al., 2016).

Less frequently, eyelid tremor, nystagmus and abnormal saccadic lateral conjugate eye movements have also been reported in some cases of chronic intoxication (El-Sherbeeny et al., 2006; Rustagi and Singh, 2010; Fowler and Zalups, 2022).

## 4 Conclusion

In summary, mercury intoxication is a major public health concern and patients suffering from systemic mercury poisoning, in addition to central nervous system damage, may exhibit a direct ophthalmic involvement, mainly as an ocular surface disease and targeting primarily the inner and outer retina with secondary impairment of the optic nerve. It also has a potential cataractogenic effect, however more studies are needed to confirm this hypothesis. Ophthalmic changes may lead to potential irreversible damage to the visual system, hence raising people awareness and leading to massive interventions to reduce major sources of human mercury exposure according to the Minamata Convention on Mercury by shifting energy production from coal burning to clean energy, eliminating gold and mercury mining, and phasing down of mercury use in some products or processes.
